# United Kingdom health research analyses and the benefits of shared data

**DOI:** 10.1186/s12961-016-0116-1

**Published:** 2016-06-24

**Authors:** James G. Carter, Beverley J. Sherbon, Ian S. Viney

**Affiliations:** Medical Research Council, Polaris House, North Star Avenue, Swindon, SN2 1FL United Kingdom

## Abstract

**Background:**

To allow research organisations to co-ordinate activity to the benefit of national and international funding strategies requires assessment of the funding landscape; this, in turn, relies on a consistent approach for comparing expenditure on research. Here, we discuss the impact and benefits of the United Kingdom’s Health Research Classification System (HRCS) in national landscaping analysis of health research and the pros and cons of performing large-scale funding analyses.

**Methods:**

The first United Kingdom health research analysis (2004/2005) brought together the 11 largest public and charity funders of health research to develop the HRCS and use this categorisation to examine United Kingdom health research. The analysis was revisited in 2009/2010 and again in 2014. The most recent quinquennial analysis in 2014 compiled data from 64 United Kingdom research organisations, accounting for 91% of all public/charitable health research funding in the United Kingdom. The three analyses summarise the United Kingdom’s health research expenditure in 2004/2005, 2009/2010 and 2014, and can be used to identify changes in research activity and disease focus over this 10 year period.

**Results:**

The 2004/2005 analysis provided a baseline for future reporting and evidence for a United Kingdom Government review that recommended the co-ordination of United Kingdom health research should be strengthened to accelerate the translation of basic research into clinical and economic benefits. Through the second and third analyses, we observed strategic prioritisation of certain health research activities and disease areas, with a strong trend toward increased funding for more translational research, and increases in specific areas such as research on prevention.

**Conclusions:**

The use of HRCS in the United Kingdom to analyse the research landscape has provided benefit both to individual participatory funders and in coordinating initiatives at a national level. A modest amount of data for each project is sufficient for a nationwide assessment of health research funding, but achieving coverage of the United Kingdom portfolio relies on sourcing these details from a large number of individual funding agencies. The effort needed to compile this data could be minimised if funders routinely shared or published this information in a standard and accessible way. The United Kingdom approach to landscaping analyses could be readily adapted to suit other groups or nations, and global availability of research funding data would support better national and international coordination of health research.

**Electronic supplementary material:**

The online version of this article (doi:10.1186/s12961-016-0116-1) contains supplementary material, which is available to authorized users.

## Background

Health research seeks to address some of the most economically significant challenges facing society. In the United Kingdom, the Department of Health (England) has estimated costs of more than £5 billion per year to the National Health Service (NHS) [1] and £27 billion to the economy [2] due to obesity, with almost 1 in 4 adults and around 15% of children being obese [3]. In 2014, the United Kingdom Alzheimer’s Society published a major study on the social and economic impact of dementia in the United Kingdom, which estimated that there will be 850,000 people living with dementia in the United Kingdom by 2015 and that dementia already costs the country £26 billion a year [4].

Therefore, changes to health service delivery and healthcare, informed by health research, has the potential to bring substantial economic and wider societal impacts. In addition, publically-funded health research is an important attractor for investment from a global, research-intensive pharmaceutical industry which in turn can bring additional associated spill-over benefits [5]. A recent analysis of the impact case studies submitted via the 2014 United Kingdom Research Excellence Framework exercise identified £2 billion of health gain from 11 such impacts alone in the period 2008–2012 [6]. Investment in health research is therefore widely regarded as generating positive economic returns [7], but estimations of these returns are often highly caveated and, indeed, the means to measure such returns require further development [8].

One challenge to obtaining better estimates of the benefits from health research is that health research is supported by a broad range of public and charitable funders working alongside the pharmaceutical industry, and thus details about the inputs into the system are not available in one place. Collaboration between these sectors are common, but each sector has its own strategic plans and policies through which funding is assigned. To co-ordinate activity across funders, with the aim of maximising impact, knowledge of the funding landscape (which research activities are funded, where and by which funder) is needed.

Funding organisations are acutely aware of the benefits of co-ordinating resources and supporting complementary, rather than competing, research. Enhanced coordination has the potential to identify new strategic investment opportunities, by highlighting areas of unmet need or prior under-investment.

However, providing a coordinated national approach to health research has three key requirements:

1. A committed partnership between health research stakeholders that create and oversee cooperative ventures.
2. A mechanism to determine the nature of health research funding on a national level which can be replicated over time.
3. A common classification system to uniformly identify types of health research being funded across a highly diverse range of funder types and research portfolios.

In the United Kingdom, processes have been in place for the last decade to coordinate health research on a national scale, to the benefit of the global research effort. This process was begun in 2004, with the establishment of the United Kingdom Clinical Research Collaboration (UKCRC) [9], a partnership between United Kingdom research funding bodies, the NHS, Government and other regulatory bodies, the biomedical industry, and patients. The UKCRC’s core aim was to “*re-engineer the clinical research environment in the UK, to benefit the public and patients by improving national health and increasing national wealth*” [9].

Part of this remit included the development and application of the Health Research Classification System (HRCS) [10], a bespoke system to analyse the United Kingdom’s health research landscape. Over the last decade, the HRCS has been used consistently within the United Kingdom, culminating with the publication of the third quinquennial analysis in August 2015. We are therefore now presented with the unique opportunity to show how the development of the HRCS and its application within the United Kingdom has influenced funding policies over the past 10 years. In particular, we wish to discuss how the collaboration between different organisations to sharing funding data has changed the nature of United Kingdom health research, with implications not just for the future of the country’s science base but for applications of classification systems as policy tools on a global scale.

## Methods

### The Health Research Classification System

In 2004, the UKCRC, via a dedicated secretariat, both developed the HRCS and conducted the first United Kingdom-wide analysis. The HRCS is a two-dimensional coding system, where each award is coded based on both the disease of focus (‘Health Category’) and type of research undertaken (‘Research Activity’). Health Categories are built from the WHO International Classification of Diseases [11], whilst Research Activities are developed from the Common Scientific Outline [12], originally developed by the United States National Institutes of Health National Cancer Institute and now in use by the International Cancer Research Partnership. The combination of the two coding arms allows a full breadth of coverage across all types of health research.

### Data collection, coding and analysis

The elements of each individual United Kingdom Health Research Analysis are explained in more detail within the published reports [13]. Here, we present a brief summary of each report’s methodology, which highlights the key details, the developments made and changes in practice from 2004/2005, through 2009/2010 and to 2014. Further information is also available in our Additional file 1: Supplementary methodology.

All three analyses required funders to submit award data via a common dataset of information consisting of a small amount of simple award information, namely basic Principle Investigator (PI) details such as Institute and Location, award type, amount and duration, alongside the award title and abstract, required for coding. The first United Kingdom Health Research Analysis (2004/2005) [14] brought together the 11 largest United Kingdom research organisations providing 9901 awards to a central database of funders’ research portfolios. The second analysis (2009/2010) comprised 12 research organisations providing 11,482 awards and the third analysis (2014) contained 17,021 awards from 64 research organisations.

To provide HRCS coding, the UKCRC Secretariat provided a complete classification check for the first analysis with each award independently coded twice (“dual coded”) then reviewed and standardised for inclusion in future HRCS training literature. Subsequent analyses relied on internal staff trained in HRCS to provide coding for awards, or via the use of dedicated external independent coders (“contract coders”). The dual coded quality control (QC) process was absent from the 2009/2010 analysis, but was replicated in the 2014 analysis on over 30% of awards.

## Results

### Analysis results: 2004/2005 to 2014

**Fig. 1:**
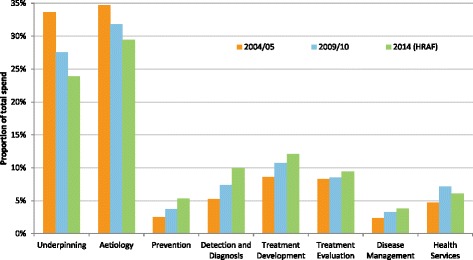
Note (Table 1 and Figure 1): Changes in proportion of research activity spend from the United Kingdom Health Research Analyses 2004–2014

The original United Kingdom Health Research Analysis (2004/2005) accounted for £950 million of spend (£1.19 billion in 2014 real terms^a^) (Table 1). Segregation by research activity showed the majority of funding (£816 million, 68.6%) was allocated to basic research types ‘Underpinning’ and ‘Aetiology’. Assessment of health categories showed ‘Generic Health Relevance’, used to classify awards relevant to all diseases and conditions or to general health and well-being, received the most funding (£300 million, 25%) followed by ‘Cancer’ (£242 million, 20%). The study also compared health category findings with United Kingdom burden of disease, via the WHO Disability Adjusted Life Years (DALYs) 2002 estimates [15], and assessed the geographical distribution of United Kingdom research funding. The differences seen in these health category comparisons over time have been relatively small versus the shifts in research activity spending (Table 1 and Fig. 1).

**Table 1:** Funding distribution by research activity for United Kingdom Health Research Analyses in 2004/2005, 2009/2010 and 2014

HRCS Research Activity Group	2004/2005		2009/2010		2014 (HRAF)		Difference in 2014 (£)		Difference in 2014 (%)	
	Value (£m)	%	Value (£m)	%	Value (£m)	%	vs. 04/05	vs. 09/10	vs. 04/05	vs. 09/10
1 Underpinning	401.7	33.6%	488.7	27.6%	453.5	23.9%	51.8	–35.2	–9.7%	–3.7%
2 Aetiology	414.4	34.7%	563.1	31.8%	558.4	29.4%	144.	–4.7	–5.2%	–2.3%
3 Prevention	29.6	2.5%	66.4	3.7%	101.5	5.4%	71.9	35.2	2.9%	1.6%
4 Detection and Diagnosis	62.9	5.3%	129.9	7.3%	189.0	10.0%	126.1	59.1	4.7%	2.6%
5 Treatment Development	102.9	8.6%	189.3	10.7%	228.8	12.1%	126.0	39.6	3.5%	1.4%
6 Treatment Evaluation	99.0	8.3%	151.5	8.5%	179.0	9.4%	79.9	27.5	1.1%	0.9%
7 Disease Management	27.7	2.3%	57.3	3.2%	71.4	3.8%	43.7	14.1	1.4%	0.5%
8 Health Services	56.2	4.7%	126.1	7.1%	114.9	6.1%	58.7	–11.2	1.4%	–1.1%
Grand Total	1194.3	100%	1772.3	100%	1896.5	100%	702.1	124.2	0.0%	0.0%

Note (Table 1 and Figure 1): The 2004/2005 analysis did not include Arthritis Research UK, so data from this charity was added retrospectively in comparisons in the 2009/2010 analysis. The 2014 analysis included contributions from 64 research organisations, however, to allow comparable data with previous analyses, only the Health Research Analysis Forum (HRAF) group funders (original 11 plus Arthritis Research UK) are included here. Award values are in 2014 real terms, extrapolated from original spend using GDP deflator data as of December 2014. See the United Kingdom Health Research Analysis 2014 main report for more details

The 2004/2005 analysis contributed to a landmark United Kingdom Government review of health research spending conducted by Sir David Cooksey in 2006 [16]. This review was intended as a means to advise the Government on the best design and institutional arrangements for public health funding in the United Kingdom. Data from the HRCS analysis were key components in assessment of the current status of United Kingdom health research upon which suggestions for future funding policy could be made.

The key recommendation from the Cooksey Review was to strengthen translation of research into health and economic benefits. The Cooksey report noted the UKCRC analysis showed two thirds of research funding is invested in basic science, and that, while the reviewers supported sustained funding in this area, they recommended “*that future increases in funding should be weighted towards translational and applied research until a more balanced portfolio is achieved*” [16].

Using the 2004/2005 analysis as baseline, the second United Kingdom Health Research Analysis (2009/2010) showed that funding for health research from the United Kingdom public and charity sectors had increased by 50% in real terms, with £1.77 billion in real terms spend accounted for. An overall increase in total spend meant that every research activity saw a real terms increase in funding, however, the proportion spent on ‘Underpinning’ and ‘Aetiology’ fell (by 6% and 2.9%, respectively) in favour of increases in the remaining six groups, particularly in the translational areas of ‘Detection and Diagnosis’ (+2.0%) and ‘Treatment Development’ (+2.1%).

In addition to assessment of HRCS classifications, both the 2009/2010 and 2014 reports included an estimation of total United Kingdom expenditure on health-relevant R&D using sources, such as the United Kingdom Office for National Statistics data on Gross Expenditure on Research and Development. The estimation in 2009/2010 was £9.28 billion, with 52% from the private sector, meaning the £1.77 billion included in the core analysis constituted approximately 40% of the total public/charity-funded health research. In comparison, the 2014 analysis estimation of total United Kingdom Health R&D expenditure was £8.5 billion with 48% from the private sector. Therefore, the £3.01 billion compiled for the 2014 analysis constituted approximately 91% of all public/charity-funded health research in the United Kingdom.

The third HRCS analysis for 2014 shows some growth in health research spend, but at a greatly reduced rate; the Compound Annual Growth Rate (i.e. the mean annual growth rate), was 4.8% over the 10 years of this analysis period (2004 to 2014), but was 8.2% in the first 5 years (2004 to 2009) and only 1.4% in the second 5 years (2009 to 2014); substantially lower than the United Kingdom growth in GDP or growth in the consumer price index, which had a 2% and 2.6% Compound Annual Growth Rate,^b^ respectively, over the same period. This low real-terms growth in expenditure over the last 5 years is consistent with data from the Association of Medical Research Charities in 2010/2011 [17] and 2013/2014 [18], and the United Kingdom Government decision to provide level funding for research via the research councils in 2010 [19]. In examination of the research activity distributions, we saw a 5 year real-terms decrease in funding for ‘Underpinning’ and ‘Aetiology’ (–£35 million and –£4.7 million, respectively), although total spend was still higher in real terms than in 2004/2005.

The proportion of spend for ‘Underpinning’ and ‘Aetiology’ had also decreased over this second 5 year period (–3.7% and –2.3%, respectively) meaning an overall reduction in proportional spending from 69% to 50% over the full 10 year reporting period. The decreases in proportional funding to basic research between 2009 and 2014 is counterbalanced with a further increase in proportional funding for ‘Prevention’ (+1.6%), ‘Detection and Diagnosis’ (+2.6%) and ‘Treatment Development’ (+1.4%). These changes are displayed in Fig. 1.

**Fig. 2:**
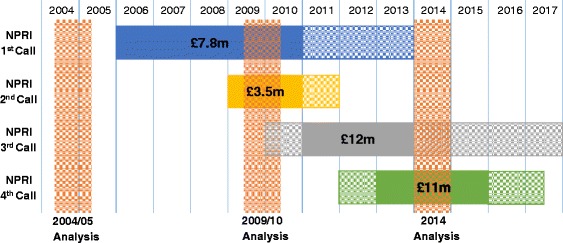
Timeline of National Prevention Research Initiative (NPRI) funding calls and United Kingdom Health Research Analysis reporting periods. Horizontal Bars indicate the start and end of NPRI funding calls. Bold sections indicate the average start and end dates for awards in each call period. Vertical Bars show the United Kingdom Clinical Research Collaboration Health Research Classification System Reporting periods (financial year for 2004/2005 and 2009/2010, calendar year for 2014). Adapted from the Medical Research Council’s NPRI Review 2015 [20]

Of interest is the contribution that strategic initiatives have made to specific areas such as prevention research. Across the 10 year reporting period, ‘Prevention’ research spending more than tripled in real terms (£29 million to £101 million) with twice the proportion of total spend (2.5% in 2004, 5.4% in 2014). The National Prevention Research Initiative (NPRI) was launched in October 2004 as a joint funding capacity building venture between 14 government departments, research councils and medical charities to support prevention research for cancer, diabetes and heart disease via risk reduction/health behaviour. The initiative has recently been evaluated by an independent expert review panel which concluded that the NPRI had successfully strengthened United Kingdom public health prevention research [20]. The process consisted of four funding phases (Fig. 2). Between the first and second UKCRC analyses, the first two funding calls were active, while third and fourth call projects would be active in the 2014 UKCRC report. A total of £34 million was committed to the NPRI over the four calls. However, it is important to note that this would only account for roughly half the increase in funding seen between 2004 and 2014. The NPRI therefore represents the major, but not the exclusive, policy initiative that has built up the level of prevention research in the United Kingdom.

The increases over the last decade in ‘Detection and Diagnosis’ and ‘Treatment Development’ suggest that the initiatives recommended by the Cooksey Review and the subsequent strategic planning by Office for Strategic Coordination of Health Research have been realised. In the 10 year reporting period, both research activity areas received £126 million more in real terms spend and increased their share of overall spend by 3.5% and 4.7%, respectively. However, it is important to note that the Cooksey review focussed on the Government funding for health research, principally via the Medical Research Council and NIHR. Charitable funders support more than 40% of both ‘Detection and Diagnosis’ and ‘Treatment Development’ spending. Indeed, individual funder comparisons show the three largest United Kingdom charitable funders, British Heart Foundation, Cancer Research UK and Wellcome Trust, had the largest proportional gains in these two research activities. While the Cooksey Review recommended increased collaboration with other research stakeholders, these stakeholders have also changed their policies to improve translational research. For example, the Wellcome Trust’s Innovation Division, established in 2003, is specifically tasked with promoting translational health research [21].

## Discussion

### Further application and development

In addition to the main HRCS analysis series, the classification system has been applied by other United Kingdom funders for internal reporting and further analyses. The full details of the classification system and data collected for the analyses are freely available via the HRCS website [10] and have been used extensively [22–26]. The majority of large funders also integrate HRCS coding into their portfolio management systems, allowing HRCS to be used for internal assessments and for answering policy questions and/or freedom of information requests. Data from HRCS coding features regularly in the Medical Research Council annual reports and appear on public award databases, including the Research Council United Kingdom’s Gateway to Research [27] and NIHR Evaluation, Trials and Studies (NETS) project portfolio [28].

In 2011, the European Medical Research Councils (EMRC, now SRG-MED) and European Science Foundation (ESF) compared a range of research classification systems, including OECD Frascati, Medical Subject Headings (MeSH), Australian and New Zealand Standard Research Classification and United States National Institutes of Health Research, Condition and Disease Categorization (RCDC) system [29]. The EMRC and ESF recommended system was HRCS, with the suggestion for cross-Europe adoption as a portfolio analysis tool [30], particularly as there was interest in performing joint European analyses to comment on policies aimed at development of the European Research Area. The HRCS has since been trialled or adopted internationally in Ireland, Sweden, Norway, Switzerland, Canada, Hong Kong and Singapore.

### Classifying award data

Categorisation of research awards is a pre-requisite for monitoring funding portfolios and allows future planning of new initiatives and strategies. Fundamental to any organisation that funds research is the ability to answer questions about what they have funded and where. As a result of this business need many research organisations use bespoke internal systems to classify award data. The HRCS could be considered just one example amongst many classification approaches and, while it has been successfully implemented in the United Kingdom, its usage and suitability for other organisations will vary depending on the research foci, funding organisations and countries involved.

There are, however, two key criteria which gives the HRCS the potential to become more widely accepted for health research. Firstly, the HRCS was adapted from pre-existing, international classification systems (i.e. International Classification of Diseases 10 & Common Scientific Outline) to ensure coverage of a full range of diseases and research activities and to allow alignment with other systems that use or are based on these systems (such as WHO Global Burden of Disease DALY data [31], or NHS England programme spend data [32]). However, while other classification systems, like MeSH and RCDC, use extensive medical research thesaurus terms, HRCS codes were specifically restricted with the explicit aim to be used at a higher aggregate level and be more useful for strategic national analysis. The design of the HRCS as a landscaping tool can lead to a perception that it lacks granularity. It is true that a more nuanced method is required to segregate within HRCS Health Categories; for example, to separate studies of Alzheimer’s from the overarching HRCS Health Category of ‘Neurological’. However, the additional costs in staff time to apply further levels of coding to a broad landscaping analysis is considerable and somewhat defeats the purpose of maintaining classifications at the wider strategic level. Indeed, it is the principle of universal application with restrained classification choices that is the greatest strength of the HRCS, hence the recommendation by the EMRC/ESF above other comparator classification systems.

Secondly, the HRCS analysis demonstrated that a small number of data fields for each award (i.e. Title, Abstract, Start & End Dates, Value, Location) are sufficient for a nationwide assessment of health research funding and can be successfully implemented by a diverse collection of research organisations for mutual benefit. This approach was feasible in practice and could be used both to monitor the effect of policy changes within individual organisations and their cumulative effect on the national funding strategy.

### Costs and benefits of health research reporting

Management of the UKCRC exercise depends on a number of components. Firstly, sufficient resources need to be available to initiate, manage, and follow-up the analyses. It is difficult to accurately estimate the full cost of the three UKCRC analyses as resources have been contributed across a number of organisations and the management of the report has changed considerably in the last 10 years of reporting. The first report (2004/2005) required substantial support, including significant time taken to convert grant details held on paper to electronic records, and a full QC exercise of all coding. The second report (2009/2010) included more detailed analysis, such as assessment of investment in infrastructure and estimation of total United Kingdom health research expenditure. However, the second analysis did not include a dedicated project management resource to co-ordinate work across a large number of funding agencies, and did not include any dual coding of awards for QC. The third analysis in 2014 engaged a part-time project manager, involved more funders, more awards, a larger proportion of coding was performed internally (rather than contracted out) than previous analyses, and a third of awards were dual coded for QC purposes.

However the estimated costs of each exercise (summarised in Table 2), alongside our anecdotal experience of producing this reporting series, suggests that funder-led practices will reduce costs over time, but that the efforts of individual funders benefit considerably from some dedicated central coordination support. Our current estimates show that changes in practice over time could reduce the cost per award to less than one third of the original analysis.

**Table 2:** Breakdown of estimated costs for United Kingdom Health Research Analysis reporting

Report	No. of Awards	Dedicated Staff	Dedicated Costs (staff, contract coding, etc.)	Internal Funder Estimates (Cost)	Approximate Report Cost Total	Total Cost per Award
2004/2005	9900	4	£185,600	£6100	£191,700	£19.36
2009/2010	11,500	0	£54,200	£32,500	£86,700	£7.54
2014	17,000	0.5	£42,000	£39,000	£81,000	£4.75

These estimations are based on the known variables/costs including contract coder expenses, dedicated salaries and report design costs. Where costs are not easily quantified, primarily internal staff time used, we provide estimates based on time for coding (~12 mins per award) required, portfolio extraction time for each participating funder and time requirements for report construction (data collection and cleaning, analysis and report writing/publication). See Supplementary Methods for further details. The 2004/2005 & 2009/2010 costs are extrapolated from original spend estimates using GDP deflator data as of December 2014. See the United Kingdom Health Research Analysis 2014 main report for more details

It is important to note that these project costs, and the costs associated with changes in funder practices to establish routine coding, may be somewhat off-set by the re-use of data for internal reporting, informing on other funding policies and benchmarking for individual intra-funder award schemes. The 2014 analysis has already proven more useful than the 2009/2010 analysis, simply because it was possible to complete it sooner after the end of 2014, and so the information within the report had greater immediate currency. Furthermore, the value of the landscaping reports themselves will continue to grow with the full underlying datasets made freely available for re-use. Despite these long-term benefits, reduction of costs is an important consideration for the future of HRCS. One potential resolution to this issue would be the development of automated coding, and at the time of writing the company UberResearch [33] was testing a promising approach for this. Using computer algorithms to apply HRCS would clearly reduce the significant human resources needed for routine coding, allowing for larger and/or more frequent analyses. However, the nuances of interpreting project descriptions and the language used to define project aims are likely to continue to require human input to ensure such algorithms can be adapted to changes in the language used in abstracts and developments in research fields. Unsurprisingly, it is clear that human coders vary in the way that they apply HRCS categories, despite minimising this variability through training and guidance. This variation can be corrected in part by coding awards multiple times, the objective of the QC process; however, as noted above, this adds significantly to the costs of the exercise. Automated coding has the potential to offer a consistent and very low cost coding approach, which could be adapted and retrospectively applied as requirements for coding change over time.

### Availability of portfolio information

The ability to obtain the necessary data on the awards funded from a large range of different research organisations is essential for landscaping analyses. In the past, the only way to obtain this information was to approach each funding organisation. Funders are beginning to provide details of their health research portfolios openly available in a form that can be systematically re-used at the award level. Within the United Kingdom, several funders now make this information publicly available as a way of accounting for the support they receive from the public. Yet, many research funders are still hesitant in making all award information publically available.

It is important to note that details of the studies supported by the private sector, which constitutes nearly half the United Kingdom’s health research and development expenditure, are not available. Due to issues of commercial sensitivity this information is unlikely to ever be publicly available at the level of individual projects. Thus, even an established landscaping project such as the UKCRC analysis series capturing 91% of public/charitable funding is still only representative of half (52%) of national health research spending [34].

## Conclusions

Given the clear benefits of better coordination of health research, there are efforts being made to compile public and charity funding information at national and international levels. International strategic collaborations such as the Heads of International Research Organisations [35] and the WHO’s World Health Assembly [36], aim to promote national, regional and global coordination of health research. In addition, efforts have already been made to establish a comprehensive international dataset of awards such as the WHO global health research and development observatory [33] and National Institutes of Health World RePORT system.^c^ Just as the United Kingdom HRCS health research analysis has proved highly beneficial, more comprehensive international databases of funding data would be invaluable to funding organisations and researchers working in the field of health research policy. There may also be benefits to the wider biomedical research community. Submitting articles for publication, compiling online CVs, completing institutional or other records of output and other administrative activities is time consuming, therefore having a single national or international record of ‘who funds what where’ in which grant/award details can be looked up, selected and transferred, rather than re-entered, would be of considerable importance.^d^ Similar to DOIs for research papers and ORCIDs for researchers, a single source of grant identifiers would support funders and researchers alike to align the outputs of research with the funding that supports it.

From our experience with the UKCRC report series, we believe that funders are increasingly seeing the benefit to sharing their portfolio data, both to their individual organisations, and collectively for strategy development at a national level. Open data from funders would allow easier benchmarking between research organisations, easier comparison of research strategies and maintaining competitiveness. In addition, research funders could identify potential partner organisations with similar or complimentary interests, more readily identify appropriate peer reviewers, and streamline researcher recruitment and funding assessments. Furthermore, shared, comparable award information will also benefit researchers, providing invaluable data for ‘science of science policy’ research and a key resource to link research funding with researcher outputs.

## Endnotes

^a^For comparison purposes, previous analysis figures are expressed in real terms (i.e. 2014 prices) using the UK GDP deflator data as at December 2014. Full details of the calculations can be found in the Methods chapter. https://www.gov.uk/government/statistics/gdp-deflators-at-market-prices-and-money-gdp-december-2014-quarterly-national-accounts. Accessed 10 June 2015.

^b^GDP and CPI data based on CAGR calculations from ONS GDP between 2010 and 2014; https://www.ons.gov.uk/economy/grossdomesticproductgdp/timeseries/abmi and http://webarchive.nationalarchives.gov.uk/20160105160709/http://ons.gov.uk/ons/datasets-and-tables/data-selector.html?cdid=D7BT&dataset=mm23&table-id=1.1, respectively, Accessed 05 Mar 2016.

^c^World RePORT is an illustrative mapping database system designed by the US National Institutes of Health (NIH) to facilitate communication and coordination of biomedical research funded by major government agencies and philanthropic organizations around the world. https://worldreport.nih.gov/index.cfm. Accessed 05 Mar 2016.

^d^Services such as the Europe Pub Med Central grant finder http://europepmc.org/grantfinder, or the ÜberWizard for ORCID provided by UberResearch Ltd. http://www.uberresearch.com/orcid-wizard/ are examples of the first datasets/processes to address part of this issue.

## Abbreviations

DALY, Disability Adjusted Life Years; EMRC, ESF Scientific Review Group for the Biomedical Sciences (SRG-MED) (formerly European Medical Research Councils, EMRC); ESF, European Science Foundation; HRCS, Health Research Classification System; MeSH, Medical Subject Headings; NHS, National Health Service; NIHR, National Institute for Health Research; NPRI, National Prevention Research Initiative; QC, Quality Control; RCDC, Research, Condition and Disease Categorization; UKCRC, United Kingdom Clinical Research Collaboration

## Additional file

Additional file 1: Supplementary Methods. (DOCX 16 kb)
