# Supplementary material for: United Kingdom health research analyses and the benefits of shared data
Source: Health Res Policy Syst. 2016 Jun 24;14:48. doi: 10.1186/s12961-016-0116-1 (PMC4919875; doi:10.1186/s12961-016-0116-1)
Supplement: Additional file 1: — Supplementary Methods. (DOCX 16 kb) [file 12961_2016_116_MOESM1_ESM.docx]

# Additional file 1: Supplementary Methods

The detailed methodology used in each individual UK Health Research Analysis is explained in more detail within the published reports^[[1]](#endnote-1)^. It is not our intention to simply replicate this information, but instead show how a standardised methodological approach was used across our ten year reporting period to allow comparable analysis of the UK health research portfolio over time.

### Administrative organisation of HRCS reporting and estimating analysis costs

The first UK Health Research Analysis (2004/05) required four dedicated UKCRC secretariat staff working full time, with additional support from participating organisation staff. Only 5 of 11 participating funders maintained electronic award records and 4660 abstracts (48% of total) were imported manually. Completion of the analysis and report took 18 months at an estimated cost of £150,000, or £185,600 in real terms. Note that in this paper, analysis spending is reported ‘in real terms’ meaning 2014 prices, which matches the most recent UK Health Research Analysis 2014. This adjustment uses UK GDP deflator data from the ONS, as of December 2014.

The second UK Health Research Analysis (2009/10)^[[2]](#endnote-2)^ followed a shift in UKCRC policy from centralised resources to a partner-led model. Coordinated via a working group, the Health Research Analysis Forum (HRAF), HRCS coding was carried out directly by the twelve participating organisation with central database management by the Medical Research Council (MRC). A total of 11,482 awards were collected over a period of 12 months with total project time to publication extending over 18 months. Without a dedicated secretariat, approximately £50,000 of additional contract coding was required. The project cost of this new partner-led model, primarily the participating funder internal staff man hours required to complete coding etc., are difficult to estimate. Based on average coding time per award and with the addition of data analysis and report production, we estimate the combined internal funder project cost at £30,000, making the overall cost of the 2009/10 analysis £80,000 (£86,700 in real terms).

The current HRCS analysis (2014)^[[3]](#endnote-3)^ continued the HRAF coordinated model with the addition of a part time dedicated project manager. This ring-fenced effort allowed for efficient expansion of the analysis to a wider range of UK research funders. In total 64 organisations with 17,021 health-relevant awards were analysed over a period of 10 months. Contract coders were still employed, although with a reduced workload. Direct costs for both contract coding and project manager were approximately £42,000 with an estimated £39,000 attributed to internal funder costs.

Data collection and analysis for all three analyses used a combination of MS Excel and MS Access database management. Submissions from individual funders required conversion from a single line award information format (with separate columns for award and coding information) into a multiple line award coding suited to pivot analysis. For more details on this process, see the UK Health Research Analysis 2014, page 96 - Appendix 10: Additional Methods. Award commitment was annualised and apportioned between assigned codes to prevent double counting of analysed funds.

### *From Donation to Innovation* (2007)

In 2007, AMRC published a companion report to the first UKCRC analysis, using 2004/05 data from 29 small to medium charities in their *From Donation to Innovation* report^[[4]](#endnote-4)^. A total of 20 of these charities returned to participate in the 2014 UKCRC analysis, allowing direct comparison with their 2004/05 data. As with the larger health funders, the comparison showed a decrease in basic research (*Underpinning* -8.1%, *Aetiology* -5.3%) in favour of translational activities (*Detection and Diagnosis* +4.7%, *Treatment Development* +2.3%, *Treatment Evaluation* +3.3%).

1. All three UK Health Research Analyses are available via the HRCS website; <http://www.hrcsonline.net/pages/reports> Accessed 03/05/2016. [↑](#endnote-ref-1)
2. UK Clinical Research Collaboration (2012). *UK Health Research Analysis 2009/10*. Published online by UKCRC <http://www.ukcrc.org/wp-content/uploads/2014/03/2UKHealthResearchAnalysis-1.pdf> Accessed 06/10/2015. [↑](#endnote-ref-2)
3. UK Clinical Research Collaboration (2015). *UK Health Research Analysis 2014.* Published online by UKCRC, ISBN 978-0903730-20-4. <http://www.hrcsonline.net/pages/uk-health-research-analysis-2014> Accessed 06/10/2015. [↑](#endnote-ref-3)
4. Association of Medical Research Charities & UK Clinical Research Collaboration (2007) *From Donation to Innovation*. Published online by UKCRC and AMRC. <http://www.ukcrc.org/wp-content/uploads/2014/03/From_Donation_to_Innovation_Report_071.pdf> Accessed 06/10/2015. [↑](#endnote-ref-4)
